# Substrates in Organic Mint Cultivation: Growth, Phytochemistry and Biological Activities

**DOI:** 10.3390/plants14182886

**Published:** 2025-09-17

**Authors:** Gilcielen de Oliveira Carreiro, Hélida Mara Magalhães, Mariana Moraes Pinc, Silvia Graciele Hulse de Souza, Zilda Cristiani Gazim, Gabriela Catuzo Canonico Silva, José Eduardo Gonçalves, Odair Alberton

**Affiliations:** 1 Postgraduate Program in Biotechnology Applied to Agriculture, Universidade Paranaense (UNIPAR), Umuarama 87502-210, Brazil; gilcielen.carreiro@edu.unipar.br (G.d.O.C.); mariana.pinc@edu.unipar.br (M.M.P.); silviahulse@prof.unipar.br (S.G.H.d.S.); cristianigazim@prof.unipar.br (Z.C.G.); gabriela.canonico@edu.unipar.br (G.C.C.S.); 2 Plant Production, Research and Innovation Specialist at Krilltech, Saa Q 3, Lots 690 and 700, Brasília 70632-310, Brazil; hmaramagal@gmail.com; 3 Postgraduate Program in Clean Technologies and Cesumar Institute of Science, Technology and Innovation (ICETI), Universidade Cesumar (UNICESUMAR), Maringá 87050-390, Brazil; jose.goncalves@unicesumar.edu.br

**Keywords:** *Mentha piperita*, organic cultivation, carvone, dihydrocarveol, soil

## Abstract

*Mentha piperita* is an essential oil (EO)-producing species with high commercial relevance. Its EOs are widely used in the pharmaceutical, fragrance, food and cosmetic industries. This study evaluated alternative substrates to industrial fertilizers, aiming to enhance plant development and EO yield while reducing environmental impact and costs. Three treatments were tested: corrected soil (CS), soil with vermiculite, vermicompost, and rock powder (SVR), and soil with bovine manure (SM). Growth parameters, macro- and micronutrient content, antioxidant and enzymatic activity (PAL), EO yield, and chemical composition were assessed. The SM treatment resulted in the highest fresh and dry biomass, nitrogen content, and PAL. The SVR treatment showed higher potassium levels, shoot/root ratio, and arylsulfatase activity. EO yield was similar across treatments, but SM had the highest carvone content (74.18%). Anthocyanin concentration was higher in SM, whereas antioxidant activity, assessed by DPPH and β-carotene assays, was greater in CS (3.98% and 96.25%, respectively) and SVR (2.96% and 98.59%, respectively). CS also exhibited higher phenolic content (687.65 mg GAE 100 g^−1^). Overall, CS and SVR enhanced antioxidant potential; however, considering biomass productivity, the use of bovine manure (SM) demonstrated greater potential, making it a more advantageous and sustainable alternative for *M. piperita* cultivation compared to the other treatments.

## 1. Introduction

The genus *Mentha* comprises 25 species of herbaceous plants [1]. Among these, *Mentha piperita* is a relevant species, widely used for medicinal purposes and in industry due to its pleasant aroma, which is applied in various personal hygiene and pharmaceutical products. It also exhibits antioxidant [2], antimicrobial [3], and anti-inflammatory properties [4]. Its essential oil (EO) has high added value and may contain menthol as the major compound, which is used in various industries, including food, pharmaceuticals, cosmetics, perfumes, and fragrances [5]. This compound also shows antimicrobial properties [6] and a sedative effect [7].

Another major compound found in the EO is carvone, which also holds industrial relevance and demand to produce flavorings and aromas. Notably, carvone can be obtained naturally only through EOs [8]. The cultivation of medicinal plants in Brazil, including mint, is often carried out in a rudimentary manner. Producers typically lack access to advanced technologies, relying mostly on traditional knowledge passed down through generations [9]. Scientific studies can aid in improving the production of these plants by aiming to enhance their physical and chemical traits, as well as the yield and composition of their EOs. Furthermore, the impact of different substrates on the growth, antioxidant activity, and chemical composition of mint and other medicinal plants of economic and pharmacological importance has not been fully elucidated, making it necessary to evaluate different nutrient sources and their effects on plant characteristics.

It is important to assess alternative substrates that aim to reduce the use of industrial fertilizers, which are costly and can lead to environmental issues such as pollution, pest resistance, and decreased food security [10]. Most of these fertilizers require importation, making cultivation costs dependent on foreign market factors. In 2021, 90% of such fertilizers were imported, creating a dependence on international markets for Brazilian agriculture [11]. Among the used substrates is expanded vermiculite, which has applications in construction, engineering, chemical industries, and agriculture [12]. It is used to reduce soil density [13] and is also associated with improved nutrient availability and absorption, resulting in increased growth and yield of dry and fresh biomass [14].

Another evaluated substrate was vermicompost, which is obtained through a process known as vermicomposting, in which earthworms are used to convert organic materials into humus-like material [15]. Although vermicompost is considered to have a low nutrient content, it is rich in microorganisms that interact beneficially with plant roots, promoting growth, physiological changes, and enhanced water and nutrient retention [16].

The use of rock powder aimed to remineralize and rejuvenate the soil, improving its fertility through the direct application of various types of rocks, minerals, low-grade ores, and waste materials from different biofertilizer or biotechnology industries. It can serve as an alternative source of macro- and micronutrients such as potassium (K) [17,18]. The application of cured manure as an organic substrate is a promising strategy for mint cultivation. In addition to being a way to reuse livestock waste, it causes less environmental damage compared to synthetic fertilizers [19]. Its use can increase plant height, number of branches and leaves, resulting in greater fresh biomass production [20]. Fertilization with cattle manure has a significant influence on the production of dry aerial biomass in peppermint plants [21].

Therefore, the present study aimed to evaluate the influence of different substrate types on peppermint (*M. piperita*) in terms of biomass production, EO yield, chemical composition, and antioxidant activity in organically grown plants.

## 2. Results and Discussion

### 2.1. Soil and Substrates

Soil analysis, based on samples collected from the pots of the CS treatment and the SVR and SM substrates after 90 days of *M. piperita* cultivation, revealed great differences among treatments in terms of substrate pH, organic matter content, density, moisture, and organic carbon (C) content (Tables S1 and S2).

Soil pH plays a fundamental role in plant development. It was observed that, following liming and cultivation, all three substrates used fell within the previously cited optimal pH range of 6.0 to 7.0, which is considered ideal for *M. piperita* cultivation [22].

The use of organic fertilizers aims to restore soil fertility and health by replenishing organic matter [23]. Accordingly, plants grown in soil with cattle manure (SM) showed higher concentrations of organic matter, improving nutrition and plant growth.

The higher moisture content in SVR may have been promoted using vermicompost, which has a high capacity for retaining both water and nutrients [24]. The higher levels of mineral residues and magnesium concentration observed in SVR may be associated with the use of vermiculite and rock powder, as both are mineral sources, including magnesium, which can be released into the soil during the cultivation period and absorbed by the plants [18,25]. In plant cells, magnesium ions (Mg^2+^) play a specific role in activating enzymes involved in respiration, photosynthesis, and the synthesis of DNA and RNA, making it essential for the development of most plants [26].

The SM substrate showed a higher organic carbon content, which significantly impacts soil quality, functionality, and health [27]. Another important component found at higher concentrations in the SM substrate was nitrogen (N), resulting in a higher C/N ratio. The fraction of mineralized organic N is linearly related to the substrate’s C/N ratio and can be used to indicate the influence of the soil microbiota on the availability of these nutrients [28].

After harvesting the *M. piperita* plants, the substrates were analyzed for enzyme activity, specifically β-glucosidase and arylsulfatase (Table 1). The SM treatment showed the highest β-glucosidase activity, followed by SVR and CS, which did not differ significantly from each other. Regarding arylsulfatase activity, the SVR treatment presented the highest values, followed by SM, which did not differ significantly from SVR, while CS showed the lowest activity (Table 1).

The enzymatic activities of β-glucosidase and arylsulfatase in the substrates are useful biological indicators for assessing soil quality. The activity of β-glucosidase can be used to indicate the presence of readily available simple sugars for the microbial population in the upper soil layer. This enzyme is also associated with organic matter content in the soil and with the soil’s capacity to release nutrients from organic sources to plant uptake [29].

For β-glucosidase, the SM treatment showed activity more than twice as high as that of the other treatments, indicating a greater cycling index of phosphorus (P) and C. These results are consistent with the higher organic matter content found in the analysis of this substrate. This enzyme is also involved in cellulose degradation and glucose production, serving as an energy source for the soil microbiota and thus favoring the development of microbial biomass [29,30,31].

Arylsulfatase is related to the sulfur cycle, making sulfate (SO_4_^2−^) available to plants and promoting the mineralization of organic sulfur sources, such as amino acids [31], by breaking the bond between oxygen and sulfur [32]. The high activity of arylsulfatase in the SM substrate supports the higher SO_4_^2−^ concentration found in the substrate analysis, likely derived from the use of cattle manure. The superior arylsulfatase activity in the SVR substrate may result from a complex interaction between nutrients and minerals released into the soil via rock powder, vermicompost, and vermiculite, which promote sulfur cycling. Notably, sulfur was not found in high concentrations in the chemical analysis of this substrate, which is consistent with the target specificity of this enzyme.

### 2.2. Agronomic Characteristics of the Plant

**Figure 1:**
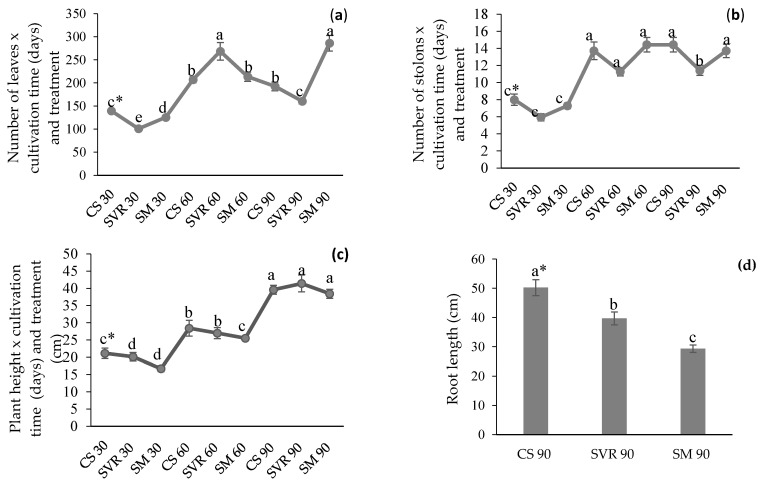
Growth of *Mentha piperita* over time (30, 60, and 90 days) under different substrates. (**a**): Number of leaves versus cultivation time and treatments. (**b**): Number of stolons versus cultivation time and treatments. (**c**): Height versus cultivation time and treatments. (**d**): Root length as a function of treatments. * Means (±standard error); followed by the same letter in the column do not differ significantly according to Duncan’s test (*p* ≤ 0.05). CS: control group (soil only); SVR: soil, vermicompost, vermiculite, and rock powder; SM: soil with cattle manure.

During the cultivation of *M. piperita*, growth characteristics were evaluated through periodic measurements of plant length, leaf, and number of stolons (Figure 1). In the first 30 days of cultivation, growth parameters were lower, increasing at 60 days and decreasing at 90 days in some treatments. The highest number of leaves was observed at 60 days for SVR and at 90 days for SM, with the SM treatment showing the highest overall average number of leaves (Figure 1a). Regarding the time factor, the 60- and 90-day cultivation periods did not show significant differences.

The average number of stolons per treatment was higher in CS and SM treatments (Figure 1b). As for plant length, all three treatments showed greater growth after 90 days of cultivation, with no significant differences observed among them (Figure 1c), and root length in the CS treatment at 90 days was higher than in the other two treatments (Figure 1d).

After harvesting the plant material and determining the fresh and dry mass (Table 2), it was observed that the SM treatment showed the highest fresh and dry aerial biomass, with 92.33 g and 29.93 g, respectively. For fresh root mass, the CS (350 g) and SM (335.75 g) treatments did not differ significantly but were superior to SVR (180.25 g). Regarding root dry mass, the SM treatment presented 58.55 g, which was 18% higher than CS (48.90 g), this latter did not differ significantly from SM, and SVR showed 39.22 g, approximately 30% lower than SM.

The cultivation period and harvest date can influence physiological aspects and growth, both due to climatic variations and the plant’s developmental stage. In this study, the highest averages for the number of leaves, stolons, and plant height were observed at 60 and 90 days of cultivation. However, when comparing data at 90 days, certain parameters declined depending on the substrate—specifically, a reduction in leaf number for SVR and in stolon number for SM. These results suggest that plant age has a significant impact on biomass yield and may be associated with the onset of senescence [33].

The greatest plant height observed at 90 days of cultivation is consistent with the findings of Desai et al. [34], who reported increased plant height, propagation, and stolon number in *M. arvensis* with longer cultivation periods, attributed to extended growth durations and favorable climatic conditions. Similarly, Al-Zyadi et al. [35] observed that *M. piperita* plants cultivated for 120 days exhibited enhanced development, including higher stolon counts and increased dry biomass of aerial parts, leaves, and roots, with no significant differences compared to plants cultivated for 100 and 140 days. The highest plant heights were recorded at 120 and 140 days. However, factors such as planting date, environmental conditions, and substrate nutrient availability must be considered when interpreting these outcomes.

The SM and SVR treatments, which showed the highest number of leaves at 90 and 60 days, respectively, compared with the control, support existing literature suggesting that plant productivity is closely linked to soil properties [36]. Thus, the use of organic fertilizers, such as cattle manure and vermicompost, may provide essential nutrients for plant growth, modifying the soil’s physicochemical properties [37], and resulting in full aerial development, as observed in this study. The first harvest could be conducted at 60 days for SVR, as maintaining these plants in the field until 90 days did not enhance growth. These findings are consistent with those reported by Mousavinik et al. [19], where the use of cattle manure in *M. piperita* cultivation was associated with increased fresh aerial biomass. Additionally, Amorim et al. [21] observed approximately a fourfold increase in dry aerial biomass in mint cultivation using cattle manure compared to other treatments.

The development of longer roots in the CS treatment, where no organic substrate was applied, may be related to the soil’s poor water retention, leading roots to grow deeper in search of nutrients and water [38,39]. In SM treatment, the root biomass was significantly higher, but with more superficial development and shorter main root length. In SVR, where vermicompost acts as a moisture retainer and vermiculite reduces soil density [13,16], root growth was reduced.

The main differences in macro and micronutrient contents among treatments were in N concentration in leaves and roots, with the highest levels found in SM (20.73 g kg^−1^ and 11.45 g kg^−1^, respectively), followed by the control group (14.05 g kg^−1^) and SVR (14.90 g kg^−1^) for leaves (Table 3 and Table 4). For roots, the control showed 7.96 g kg^−1^, and SVR 7.53 g kg^−1^. P content in SM roots was also higher at 0.85 g kg^−1^, followed by SVR with 0.56 g kg^−1^ and the control group with 0.51 g kg^−1^. Regarding K in leaves and roots, SVR showed higher concentrations, with 26.1 g kg^−1^ and 12.23 g kg^−1^, respectively (Table 3). The control had 13.45 g kg^−1^ and SM 14 g kg^−1^ in leaves, while in roots, the control had 5.72 g kg^−1^ and SM 8.85 g kg^−1^. Ca in leaves decreased in the SM treatment.

The use of cattle manure in the SM treatment resulted in a higher N concentration in the plants, promoting increases in biomass, number of leaves, and root dry mass due to the modulation of N in cellular structure and composition [40]. Nitrogen deficiency is associated with altered leaf coloration and reduced plant development and growth [41].

The higher K concentration found in SVR was attributed to the use of rock powder as an alternative source through mineralization [26]. An adequate supply of macronutrients such as N, P, and K is essential for optimal growth and development of mint species [42]. Consequently, plants receiving organic fertilization showed higher macro- and micronutrient concentrations compared to the control treatment (Table 4).

The SVR treatment showed approximately fivefold higher Fe concentrations compared to the other treatments, along with elevated Cu and Mn levels in the roots (Table 4). While these micronutrients are essential for plant nutrition, excessive concentrations can induce metabolic imbalances [43], which may have negatively affected the growth of plants in this treatment.

### 2.3. Relative Chlorophyll Index

**Figure 2:**
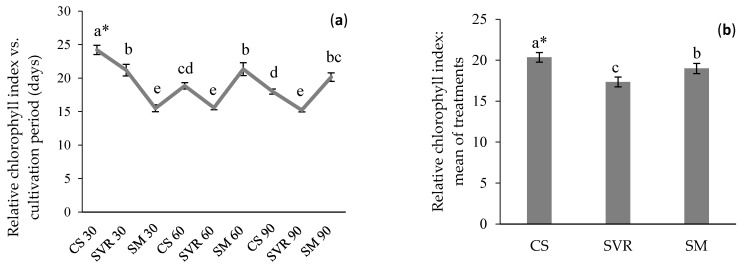
Relative chlorophyll index (RCI) of *Mentha* *piperita* vs. cultivation period (30, 60 and 90 days) and substrate. (**a**): RCI versus cultivation time and different treatments. (**b**). Mean of RCI for the different treatments. * Means (±standard error); followed by the same letter in the column do not differ significantly according to Duncan’s test (*p* ≤ 0.05). CS: control group (soil only). SVR: soil, vermicompost, vermiculite, and rock powder. SM: soil with cattle manure.

The highest RCI was observed in the CS treatment at 30 days of cultivation (24.22), followed by SM at 60 days (21.34) and SVR at 30 days (21.20) (Figure 2a). The total RCI for the CS treatment was higher overall (Figure 2b). Considering the cultivation period, the RCI was highest in the first 30 days (20.31) for all treatments, followed by 60 days (18.59) and lastly 90 days (17.80) (Figure 2b).

The relative chlorophyll index is an important physiological parameter because chlorophyll directly participates in the plant’s primary metabolism. Chlorophyll molecules are vital for transforming light energy into chemical energy through the photosynthesis process [44]. The chlorophyll content can be affected by various environmental factors, including water availability, nutrient status, light intensity, air pollution, cultivation period [45], and the presence of heavy metals [46].

In this study, the highest RCI was observed in the CS treatment at 30 days of cultivation (24.22), followed by the SM treatment at 60 days (21.34) and the SVR treatment at 30 days (21.20). These values are slightly lower than those reported by Nozzi et al. [47] for *M. piperita* cultivation, where the RCI was about 25.

### 2.4. Phytochemical and Biological Aspects of the Plant

The EO chemical compounds from the peppermint plants in the three treatments were analyzed using gas chromatography–mass spectrometry (GC-MS). The major compound in the SVR and SM treatments was carvone, present at varying concentrations. In contrast, the major compounds in the CS treatment were dihydrocarveol and carvone (Table 5 and Figure S1). Variations in the other compounds were also observed among the treatments.

In the CS treatment, which involved only corrected soil, a high proportion of dihydrocarveol (34.45%) was detected alongside carvone (31.98%) in the EO (Table 5). The presence of dihydrocarveol can be explained by microbial and/or enzymatic biotransformation processes, in which carvone, a ketonic monoterpene, undergoes reduction to a chiral alcohol. Santos et al. [76] demonstrated that filamentous fungi can convert carvone into dihydrocarveol with yields ranging from 9.5% to 100%, reaching diastereomeric excesses greater than 89%. This conversion mechanism has also been reported in bacterial systems, such as in *Rhodococcus erythropolis*, where the metabolic pathway of carveol and dihydrocarveol involves specific reductase enzymes [77]. Consequently, under conditions of lower nutrient input and higher oxidative stress, as observed in CS, secondary metabolism may favor the partial reduction in carvone, resulting in an EO with a more diversified composition of ketones and terpenic alcohols.

In contrast, the SVR and SM treatments presented carvone as the exclusive major compound, at concentrations of 51.33% and 74.18%, respectively (Table 5). This result can be attributed to a more balanced nutrient supply and the greater availability of organic matter and micronutrients, which modulate monoterpene biosynthesis. Previous studies have shown that the composition of peppermint EOs can vary significantly depending on management practices, water availability, and extraction conditions [78,79]. Under these conditions, the stability of the biosynthetic pathway leading to carvone is favored, reducing the need for alternative reduction routes. Furthermore, the higher enzymatic activity observed in SM and SVR, especially β-glucosidase and arylsulfatase, suggests greater metabolic efficiency, which may have contributed to the predominance of carvone.

The predominance of carvone in systems with organic fertilization is particularly relevant from a biotechnological and pharmacological perspective. This monoterpene is considered to have high added value by the food, cosmetic, and pharmaceutical industries and is also recognized for its antioxidant, anti-inflammatory, and antimicrobial properties [79,80]. In fact, the maintenance of carvone as the major compound in SVR and SM is close to the desirable chemical profiles reported in optimized *M. piperita* cultivations [81], in which organic fertilization not only favors productivity but also provides greater consistency in the EO chemical composition. Therefore, the results obtained demonstrate that nutritional management acts as a key factor in both modulating primary metabolism and directing the biosynthetic pathways responsible for the phytochemical quality of EO (secondary metabolites).

**Figure 3:**
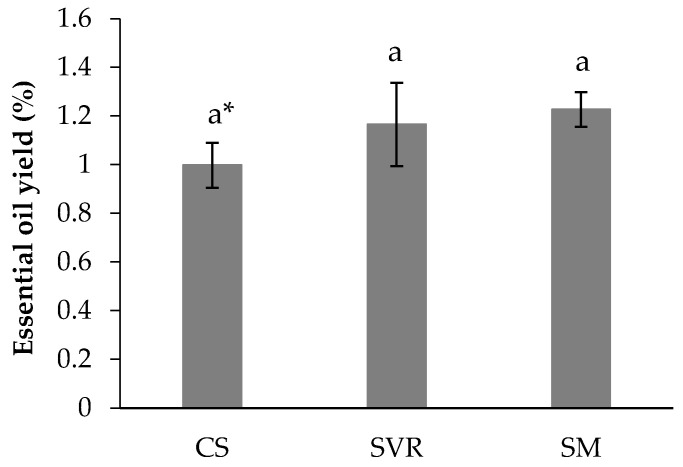
Essential oil yield (%) of *Mentha piperita* grown under CS, SVR, and SM treatments. * Means (±standard error); followed by the same letter in the column do not differ significantly according to Duncan’s test (*p* ≤ 0.05). CS: control group (soil only); SVR: soil, vermicompost, vermiculite, and rock powder; SM: soil with cattle manure.

The SM treatment showed the highest EO yield (1.23%), followed by SVR (1.17%) and CS (1.00%), the values did not differ significantly from one another (Figure 3).

The EO yields obtained from the three treatments are consistent with values reported in the literature, which range from 0.277% to 1.240% for peppermint cultivated in Egypt [82], and approximately 1.00% to 1.20% for *M. piperita* grown with cattle and poultry manure [83]. In this study, EO yield was 1.00% in the CS treatment, equivalent to 10 g of EO kg^−1^ of dry plant material. The SVR treatment yielded 11.7 g kg^−1^ (1.17%), while the SM treatment produced the highest yield at 12.3 g kg^−1^ of dry material (1.23%); the results did not differ significantly from one another (Figure 3). It is important that the SM treatment also resulted in the greatest production of fresh and dry aerial biomass, which would further increase total EO yield plant^−1^ and offer a greater economic advantage for commercial cultivation.

Although menthol and menthone are often reported in the literature as the main components of *M. piperita* EO [33,84], carvone was the major compound found in the three EO samples analyzed. The CS treatment showed 34.18% dihydrocarveol and 31.98% carvone, SVR had 51.33% carvone, and SM presented 74.18% dihydrocarveol (Table 5). However, a high content of carvone was also reported by Barros et al. [85], 58.79%, who additionally detected limonene as one of the main constituents, in agreement with our SM sample (6.35%), which was higher than in the other treatments. Similarly, Alsaraf et al. [86] reported carvone as the major compound in peppermint EO (65.21%), while Satmi et al. [87] observed 34.94% carvone and 11.20% d-limonene. In line with these findings, Rezende et al. [88] detected carvone (84.34%) and limonene (10.97%) as the major constituents in peppermint EO.

The percentage of carvone in peppermint EO can vary significantly depending on the region, and this compound is primarily responsible for the pungent and aromatic odor characteristic of the oil [86]. In addition to geographical factors, environmental conditions such as rainfall and climate also play a crucial role in influencing EO composition [89].

The EOs from the three treatments were classified as belonging to the carvone chemotype, as they contained one or more of its stereoisomers as major components [90]. Since there was no difference in the main compound among the treatments, the higher presence of carvone may be associated with environmental factors rather than differences in nutrient availability or water stress, especially considering that all treatments received identical irrigation schedules and volumes.

Carvone is a monoterpene ketone commonly found in the EOs of various medicinal plants, particularly within the Lamiaceae and Asteraceae families [91]. This compound exhibits several pharmacological properties, including antioxidant activity [92], anti-inflammatory effects [93], and anesthetic potential [94]. The carvone isomer is approved in the United States for use as an insect repellent, while D-carvone is utilized in the Netherlands as a commercial sprout suppressant for stored potatoes [95]. As carvone is exclusively obtained from natural sources, particularly peppermint EO, its demand has grown in parallel with the increasing interest in natural and plant-derived products [8].

Regarding the other major compounds identified in higher concentrations in the analyzed EOs, variations in their levels were observed. These differences may be attributed to the influence of the substrate on secondary metabolism, as plant nutrition is known to modulate the biosynthesis of secondary metabolites [96].

In agriculture, the addition of nutrients, especially N, is commonly used to increase biomass production. However, these nutrients influence not only primary metabolic processes but also the synthesis of secondary metabolites [96]. The effects of N, P, and K fertilization on the production of secondary metabolites, such as terpenes, are complex and not yet fully understood. In some cases, increased concentrations have been observed due to water deficit or increased P, while responses to N and K vary by study. For N-containing secondary metabolites, limited N availability may hinder their biosynthesis [97]. Vermicompost serves as a source of N, P, and K, supporting both plant development and secondary metabolic pathways [98]. Moreover, it contains plant growth-promoting substances such as hormones and enzymes that enhance the decomposition of organic matter in the soil, thereby increasing nutrient availability [99].

Therefore, the composition of other major EO components varied among treatments. In the CS treatment, alongside dihydrocarveol and carvone, germacrene-D (5.89%) and carveol (2.08%) were identified. In SVR, the main compounds after carvone were β-cubebene (10.34%), caryophyllene (5.92%), and β-bourbonene (5.36%). In SM, the major components following carvone were limonene (6.35%), β-cubebene (3.97%), and cis-dihydrocarvone (2.36%) (Table 5). These differences suggest that substrate composition can influence the secondary metabolite profile of *M. piperita* EOs, particularly the relative abundance of monoterpenes and sesquiterpenes.

**Figure 4:**
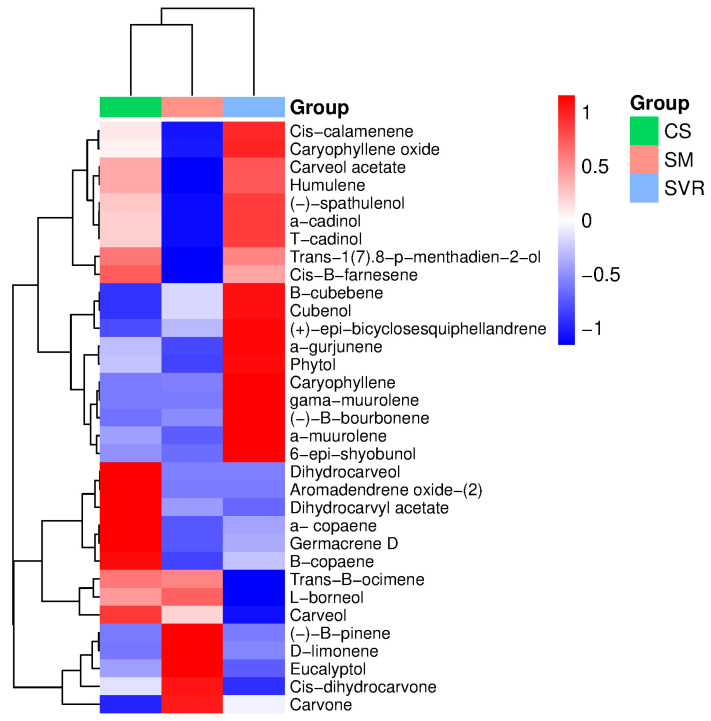
Heat map and hierarchical clustering of essential oil constituents in *Mentha piperita* grown under three substrate treatments: CS: control group (soil only); SVR: soil, vermicompost, vermiculite, and rock powder; SM: soil with cattle manure.

The heat map combined with hierarchical clustering (Figure 4) revealed distinct chemical EO profiles among treatments. The SVR treatment formed a separate cluster, indicating that the combination of vermicompost, vermiculite, and rock powder markedly altered peppermint secondary metabolism. SVR was characterized by higher relative levels of oxygenated sesquiterpenes, including humulene, (-)-spathulenol, α-cadinol, T-cadinol, and cubenol, resulting in greater diversity and intensity of these metabolites. This effect can be attributed to the balanced nutrient supply, higher cation exchange capacity, and improved water retention, conditions that favor more complex biosynthetic pathways [100].

In contrast, SM promoted the accumulation of monoterpenes such as D-limonene, eucalyptol, and carvone, while the control (CS) displayed a more restricted profile, dominated by basic monoterpenes (trans-β-ocimene and β-pinene) at lower intensities. This outcome may be explained by the initial availability of N and P from cattle manure [101]. On the other hand, the CS control showed that corrected soil alone, although sufficient for basic plant growth, did not provide the necessary nutrients and physicochemical conditions to maximize EO biosynthesis. The lower accumulation of compounds such as carvone and eucalyptol highlights peppermint’s dependence on substrates enriched with organic matter and available nutrients.

**Figure 5:**
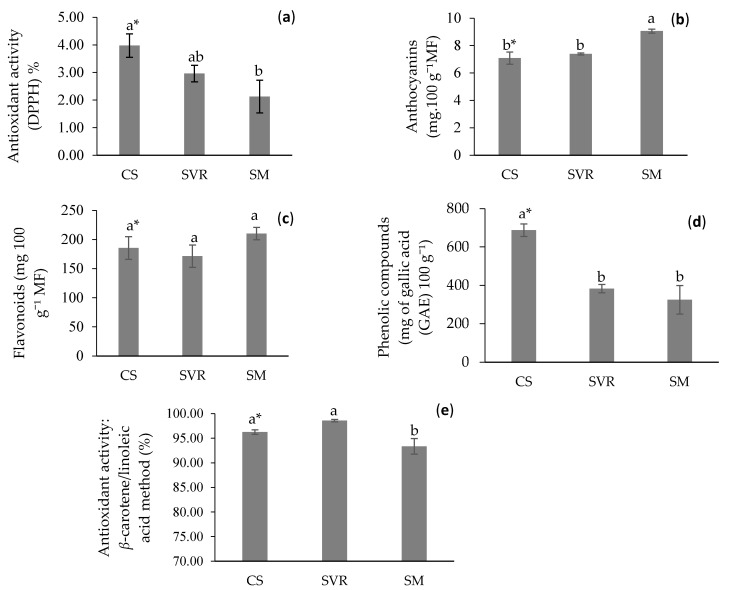
Antioxidant activity of *Mentha piperita* extracts as a function of different treatments. (**a**): Antioxidant activity of the extract using the DPPH method. (**b**): Anthocyanin content in the extract. (**c**): Flavonoid content in the extract. (**d**): Phenolic compounds in the extract. (**e**): Antioxidant activity of the extract using the β-carotene/linoleic acid method under different treatments. * Means (±standard error); followed by the same letter in the column do not differ significantly according to Duncan’s test (*p* ≤ 0.05). CS: control group (soil only). SVR: soil, vermicompost, vermiculite, and rock powder. SM: soil with cattle manure.

Regarding antioxidant activity, as evaluated using the 2,2-diphenyl-1-picrylhydrazyl (DPPH) radical scavenging method (Figure 5a), no significant differences were observed between the CS (3.98%) and SVR (2.96%) treatments. Although the SM treatment showed a lower antioxidant activity (2.13%), it was not significantly different from SVR. These results suggest that the substrate type had a limited impact on the antioxidant potential of the EOs under the conditions tested.

Antioxidant activity was also evaluated using the ferric reducing antioxidant power (FRAP) method; however, quantification was not possible as the plant extracts yielded only trace or undetectable results.

Anthocyanin quantification in *M. piperita* extract varied significantly according to the cultivation substrate (Figure 5b). The SM treatment showed the highest anthocyanin content (9.06 mg 100 g^−1^ FW), followed by SVR (7.39 mg 100 g^−1^ FW) and CS (7.08 mg 100 g^−1^ FW), with no statistically significant difference between the latter two.

Flavonoid concentrations in the *M. piperita* extracts did not differ significantly among the treatments, with values of 210.22 mg 100 g^−1^ FW for SM, 185.53 mg 100 g^−1^ FW for CS, and 171.38 mg 100 g^−1^ FW for SVR (Figure 5c).

The highest antioxidant activity found in the CS sample may be related to its higher phenolic compound content (Figure 5d), followed by the other treatments, as a positive correlation between antioxidant activity and phenolic content has been reported in *M. piperita* extracts [102]. Conversely, the lower antioxidant activity observed via the DPPH method may be associated with reduced phenolic concentrations [103].

The DPPH assay provides better responses for predominantly phenolic compounds and, to a lesser extent, for molecules with limited polarity [104]. Since the EO of *M. piperita* obtained in this study was mainly composed of monoterpenes and sesquiterpenes (L-carvone, germacrene-D, β-caryophyllene), the low activity observed in this method can be attributed to the limited hydrogen/electron-donating ability of these terpenes compared to phenolic antioxidants. The β-carotene/linoleic acid method can be particularly useful for investigating lipophilic antioxidants in plants with a high EO content, such as mint [105].

Using the β-carotene/linoleic acid method, the CS and SVR treatments demonstrated significantly higher antioxidant activity (96.25% and 98.59%, respectively) compared to SM (93.36%) (Figure 5e). In contrast, Raeisi et al. [106] reported a lower activity of 71.33% in *M. piperita* EO.

A higher concentration of anthocyanins was detected in the SM plants, which could contribute to the antioxidant activity in this group, due to the antioxidant potential attributed to this class of compounds [107]. The increased levels of anthocyanins, phenolic compounds, and flavonoids may reflect a plant response to reactive radicals generated by the substrates used, as reported by Calgaro et al. [108], who reported that the concentrations of these compounds varied depending on the treatment applied to peppermint plants grown under controlled conditions.

The production of phenolic compounds in plants often increases under conditions of limited water availability or mineral deficiency, serving as a protective response to stress [109,110]. Consequently, the lower phenolic content observed in plants cultivated with organic fertilizers may reflect improved nutrient availability and enhanced moisture retention by the substrate. Nutritional or hormonal stress can thus lead to elevated antioxidant activity [111]. In this context, the CS treatment, which received no fertilization, showed the highest antioxidant activity, likely due to the nutritional stress experienced by the plants.

Data from analyses of chlorophyll concentration, phenolic compounds, and other parameters can contribute to the development of enhanced decision-support systems in agriculture [112].

**Figure 6:**
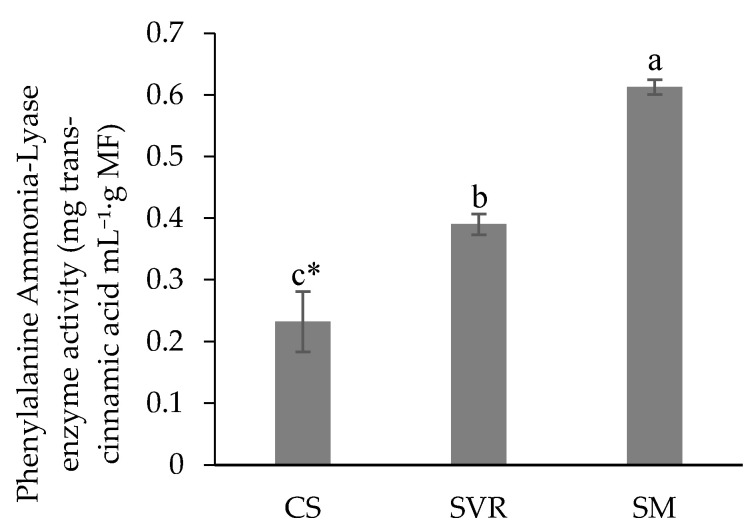
Phenylalanine Ammonia-Lyase enzyme activity from *Mentha piperita* CS, SVR, and SM extracts. * Means (±standard error); followed by the same letter in the column do not differ significantly according to Duncan’s test (*p* ≤ 0.05). CS: control group (soil only). SVR: soil, vermicompost, vermiculite, and rock powder. SM: soil with cattle manure.

Analysis of phenylalanine ammonia-lyase (PAL) enzyme activity indicated that the SM treatment exhibited the highest activity, followed by SVR, while the CS treatment showed the lowest PAL (Figure 6). The enzyme PAL is essential in the phenylpropanoid biosynthetic pathway, which leads to the synthesis of various phenolic compounds, including flavonoids [113]. It also plays a crucial role in the biosynthesis of lignin, salicylic acid, and other phenylalanine-derived metabolites [114].

In the present study, an inverse relationship was observed between phenolic compound concentration and PAL in the CS treatment. Contrary to expectations, enzymatic activity was lower than that observed in the SM treatment, which exhibited higher PAL alongside superior phenolic content. As reported by Ortega-García et al. [115], PAL enzyme activity can vary due to gene regulation at different plant developmental stages, and fluctuations in phenolic compound concentrations are not always directly proportional over time. These discrepancies reflect the complex regulatory mechanisms that govern this class of enzymes, which operate at transcriptional, post-transcriptional, and post-translational levels [116].

The variation in PAL among treatments may be associated with the developmental stage of the plants in each condition. Another plausible explanation is the regulation of enzyme expression through feedback mechanisms, potentially triggered by the accumulation of trans-cinnamic acid or other intermediate products of the phenylpropanoid pathway. In this context, the elevated phenolic content observed in the CS treatment may have induced such feedback regulation, resulting in reduced PAL. Alternatively, this downregulation could also be influenced by exogenous factors [117].

**Figure 7:**
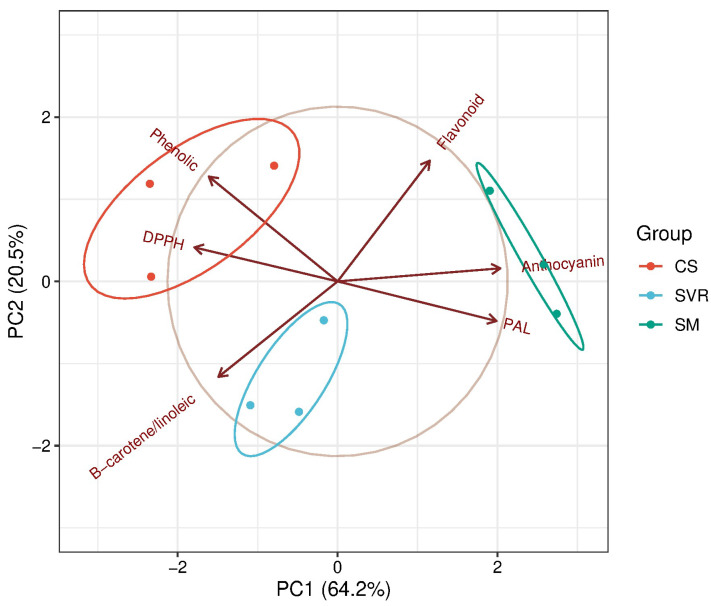
Principal Component Analysis (PCA) of antioxidant activity and phenolic compound variables in *Mentha piperita* grown under three substrate treatments: CS: control group (soil only); SVR: soil, vermicompost, vermiculite, and rock powder; SM: soil with cattle manure.

The Principal Component Analysis (PCA) analysis explained 84.7% of the total variance with the first two principal components (PC1 = 64.2%, PC2 = 20.5%), clearly separating the treatments according to their antioxidant and phenolic compound profiles (Figure 7).

The CS treatment clustered with higher total phenolics and DPPH radical scavenging activity, indicating that plants grown in corrected soil activated antioxidant mechanisms associated with phenolic accumulation. In contrast, SVR was associated with β-carotene/linoleic acid activity, suggesting that the combination of vermicompost, vermiculite, and rock powder stimulated the production of lipophilic antioxidants with strong protective capacity against lipid peroxidation. The SM treatment was characterized by higher anthocyanin and flavonoid contents, as well as increased phenylalanine ammonia-lyase (PAL) activity, indicating that cattle manure favored the phenylpropanoid pathway, thereby enhancing pigment biosynthesis.

## 3. Materials and Methods

### 3.1. Substrates and Plant Cultivation

The cultivation was conducted in the rural area of Pérola, Paraná, Brazil, at Chácara São Pedro, lot 139a, located along Corcovado Road (latitude −23.855915726060456, longitude 53.75081942333717), between October and December 2022. The soil in the cultivation area is classified as latosol [118].

The region’s climate is classified as subtropical, with an average temperature of 22.24 °C during the spring season [119]. Between October and December 2022, the average precipitation was 87.76 mm [120]. For the experiment, seedlings of *M. piperita* were used, obtained from the Medicinal Garden Herbarium of UNIPAR, cataloged under exsiccate number 2398.

Before planting, a soil analysis was conducted using samples collected at depths of 0–50 cm for physical analysis and 0–20 cm for chemical analysis [121,122] (Table S1). The soil composition was determined to be 8.00% clay, 1.45% silt, and 90.55% sand. Based on these results, liming was carried out to increase base saturation to 70%, utilizing a corrective material with a relative power of total neutralization of 85%, and applying 1.41 t ha^−1^ of limestone. The soil was previously sterilized by autoclaving for 1 h at 120 °C. Subsequently, limestone was incorporated into the soil, which was then moistened and manually turned daily for seven days to ensure uniform distribution.

The seedlings used in the experiment were commercially sourced and selected for uniformity in size, number of stolons, and leaf count. All seedlings were propagated via cuttings and were approximately 30 days old at the time of selection. Each seedling was transplanted into a 5 L plastic pot, with one plant per pot, resulting in a total of 56 pots (Six per treatment in triplicate).

Peppermint was cultivated using three different substrate treatments. In the first treatment (CS, control), only the corrected soil was used. The second treatment (SVR) consisted of a mixture of soil, vermicompost, and vermiculite in a 1:1:1 (*v*/*v*) ratio, supplemented with 200 g m^−2^ of rock powder per pot, following the manufacturer’s recommendations. The vermicompost used had the following properties: pH 7.5, total nitrogen 0.5%, cation exchange capacity (CEC) of 120 mmolc kg^−1^, CEC per carbon of 8%, organic carbon content of 15%, carbon-to-nitrogen (C/N) ratio of 30%, and a maximum moisture content of 50%. The rock powder presented the following characteristics: 5% P_2_O_5_ soluble in citric acid, 8.5% calcium, 8% total organic carbon, CEC of 225 mmolc kg^−1^, pH 6.0, and a maximum moisture content of 20%.

In the third treatment (SM), the substrate consisted of a 1:1 (*v*/*v*) mixture of soil and cured cattle manure. The moisture content of the cured manure was determined by drying samples in a forced-air oven at 65 °C until reaching a constant weight, resulting in an average moisture content of 8.98% ± 0.07%.

### 3.2. Pest Control

During plant growth, pest control (e.g., caterpillars) was performed manually by handpicking. Additionally, to support organic pest control measures, a single application of commercially sourced neem oil was applied after 60 days of growth, following the manufacturer’s guidelines.

### 3.3. Enzymatic Analysis of Substrates

After 90 days of cultivation, substrate samples from the three treatments were collected, refrigerated, and subsequently analyzed in triplicate for the enzymes β-glucosidase and arylsulfatase, following the methodology described by BioAS Technology [123].

### 3.4. Agronomic Characteristics of the Plant

Every 30 days, the number of leaves and stolons was determined by manual counting, and branch length (cm) was measured using a metric ruler. After 90 days of cultivation, fresh weight (g) was recorded with a digital precision balance, and the main root length (cm) was measured.

The collected plant material was washed under running water, and leaves and roots were separated. Samples were then oven-dried at 65 °C until constant weight and weighed using an analytical balance to determine dry mass (g). Subsequently, macro- and micronutrients, including nitrogen (N), potassium (K), phosphorus (P), zinc (Zn), iron (Fe), and copper (Cu), were quantified following the methodology described by Malavolta et al. [124].

### 3.5. Plant Physiological Indices

Every 30 days, the relative chlorophyll index (RCI) was measured on fresh leaves using the DUALEX SCIENTIFIC+™ device, Quick Start model (FORCE A^®^, Paris, France), according to the manufacturer’s instructions. Six replicates per treatment were conducted by positioning the device’s leaf clip around fully expanded leaves located in the middle third of each plant.

### 3.6. Phytochemical and Biological Aspects of the Plant

#### 3.6.1. Essential Oil Extraction and Chemical Characterization

After completing the experimental assays, leaves intended for essential oil (EO) extraction were placed in paper bags and stored at 25 ± 2 °C until they reached constant weight. For hydrodistillation, 16 g of dried leaves from the CS and SVR treatments, and 20 g from the SM treatment, were used [125]. The plant material was crushed and immersed in 3500 mL of distilled, deionized water. Hydrodistillation was conducted for 3 h using a modified Clevenger apparatus [126]. EO yield was calculated as the percentage ratio of the mass of EO obtained to the mass of plant material used (% *w*/*w*). The extracted EO was stored in amber glass vials wrapped in aluminum foil, sealed, and refrigerated at approximately 4 °C.

Chemical identification of the EO was performed using a gas chromatograph (Agilent 7890 B, GC/MS, Santa Clara, CA, USA) coupled to a mass spectrometer (Agilent 5977 A, GC/MS, Santa Clara, CA, USA), equipped with an Agilent HP-5MS UI capillary column (30 m × 0.250 mm × 0.25 μm) [125], and the identification was based on comparison with mass spectra from the Nist 11.0 library.

#### 3.6.2. Determination of Antioxidant Activity, Flavonoids, and Anthocyanins in the Extract

Antioxidant activity, flavonoid content, and anthocyanin levels were evaluated. Absorbance measurements for all antioxidant assays, except the β-carotene oxidation method, were performed using a UV-VIS spectrophotometer (Spectra Max Plus^®^, Molecular Devices, San Jose, CA, USA). The β-carotene oxidation assay utilized an ELISA Spectra Max Plus 384^®^ spectrophotometer (Molecular Devices, San Jose, CA, USA). Three biological replicates were analyzed in triplicate. Data were used to generate graphs, and standard deviations (n = 3) were calculated for each treatment.

Antioxidant activity was assessed using 100 mg of fresh leaves collected at the end of the 90-day cultivation period. Samples were ground in liquid nitrogen and stored at −80 °C until analysis. Four methods were employed: total phenolic compounds, 2,2-diphenyl-1-picrylhydrazyl radical (DPPH) scavenging, ferric reducing antioxidant power (FRAP), and the β-carotene/linoleic acid assay. All procedures were conducted in the absence of light. Extracts were prepared by macerating the leaves in 50% methanol and 70% acetone, then left to rest for two hours. The mixture was centrifuged at 12,000× *g*, and the supernatant was collected [127].

Total phenolic content was determined using 10% Folin–Ciocalteu reagent and 4% sodium carbonate [128], with absorbance measured at 750 nm. Results were expressed as mg of gallic acid equivalents (GAE) per 100 g of sample, based on a gallic acid standard curve (0, 10, 20, 30, 40, 50 μg mL^−1^) described by the equation y = 0.0076x − 0.02 (R^2^ = 0.996).

The antioxidant activity assay based on scavenging of the DPPH radical (60 μM) was conducted according to the method described by Rufino et al. [129]. Absorbance was measured at 515 nm every 30 min until stabilization. Results were expressed as percentage of radical scavenging activity (%RSA), calculated using the formula: %RSA = [(AC − AS) × 100]/AC. Where AC = absorbance of the control, and AS = absorbance of the sample.

The FRAP assay was performed using a reagent consisting of sodium acetate buffer (0.3 M, pH 3.6), ferric chloride (20 mM), and 2,3,5-triphenyltetrazolium chloride (TPTZ, 10 mM) [130]. Absorbance readings were taken at 595 nm, and results were expressed as mg of TROLOX equivalents per 100 g, based on a standard curve (0, 160, 320, 480, 640, 800, 1600 μmol L^−1^): y = 0.4136x − 0.2515 (R^2^ = 0.99) [131].

Antioxidant activity was assessed using the β-carotene bleaching method, which measures the spectrophotometric degradation of β-carotene induced by oxidative products of linoleic acid [132]. The β-carotene solution was prepared by dissolving 30 mg of β-carotene in 2 mL of chloroform. In a light-protected flask, 20 μL linoleic acid, 265 μL Tween 40, 25 μL of the β-carotene solution, 0.5 mL chloroform, and 20 mL of distilled oxygenated water were combined and vigorously mixed. Aliquots of 280 μL of this emulsion were transferred to ELISA plates containing 20 μL of the plant extracts. Absorbance was measured at 470 nm using an ELISA Spectra Max Plus 384^®^ spectrophotometer. After the initial reading, the plates were incubated in a water bath at 50 °C, and the absorbance was recorded again at 470 nm after 120 min. Antioxidant activity (%AA) was calculated as the percentage of oxidation inhibition using the equation: %AA = [1 − (Absc_initial − Absc_final)/(Abss_initial − Abss_final)] × 100.

Where %AA is the percentage of antioxidant activity, Absc_initial and Absc_final are the initial and final absorbance values of the control, Abss_initial and Abss_final are the initial and final absorbance values of the sample, respectively.

For the determination of anthocyanins and yellow flavonoids, the method described by Francis [133] was employed. Fresh leaves (100 mg) were weighed and macerated in liquid nitrogen. Extraction was performed using 5 mL of an ethanol–HCl (1.5 M) solution in light-protected 15 mL Falcon tubes. The mixture was centrifuged at 7000× *g* for 10 min, and the supernatant was collected. Absorbance was measured at 535 nm for anthocyanins and 374 nm for flavonoids. Results were expressed in mg per 100 g of fresh mass (FM), using a dilution factor of 5000 and the following equations: Anthocyanins = (absorbance at 535 nm × dilution factor)/98.2; Flavonoids = (absorbance at 374 nm × dilution factor)/76.6.

#### 3.6.3. Determination of Phenylalanine Ammonia-Lyase (PAL) Enzyme Activity in Leaves

The activity of the enzyme Phenylalanine Ammonia-Lyase (PAL) (EC 4.3.1.24) was determined using fresh leaves, following the protocols described by [134,135]. Calibration curves were established with six concentrations of trans-cinnamic acid (0.00, 0.04, 0.08, 0.12, 0.16, and 0.24 g L^−1^). Analyses were performed in triplicate using microplates, and absorbance readings were taken at 490 nm with an ELISA Spectra Max Plus 384^®^ spectrophotometer (Molecular Devices, San Jose, CA, USA). Enzyme activity (expressed in g L^−1^) was calculated from the linear regression equation obtained from the standard curve (R^2^ = 0.99), which relates absorbance to trans-cinnamic acid concentration.

### 3.7. Statistical Analysis

The experiments were conducted using a Completely Randomized Design (CRD), with six pots per treatment, totaling eighteen experimental units. The entire experiment was repeated three times simultaneously.

Data collected from growth parameters, physiological traits, phenolic compound content, essential oil yield, and antioxidant activity were first tested for normality using the Shapiro–Wilk test (*p* ≤ 0.05). Following confirmation of normal distribution, data were subjected to analysis of variance (ANOVA).

The standard error of the mean was calculated, and treatment means were compared using Duncan’s multiple range test at a significance level of *p* ≤ 0.05. All statistical analyses were performed using IBM SPSS Statistics^®^ version 29. The heat map and PCA analyses were performed using SRplot (https://www.bioinformatics.com.cn/en?p=1, accessed on 30 June 2025) [136].

## 4. Conclusions

Soil treatment with cattle manure (SM) demonstrated the greatest potential for promoting plant growth, as evidenced by an increase in the number of leaves, greater fresh and dry biomass of the aerial parts, higher N concentration, enhanced phenylalanine ammonia-lyase (PAL) activity in leaf extracts, and elevated activities of the β-glucosidase and arylsulfatase enzymes in the substrate.

Variations in the concentration and quantity of chemical compounds in the essential oil (EO) were observed across the three treatments; however, all samples were classified as belonging to the carvone chemotype.

Therefore, the SM treatment demonstrated greater advantages for the cultivation of *Mentha piperita* compared to the use of corrected soil alone (CS) or the combination of soil with vermiculite, rock powder, and vermicompost (SVR). Moreover, leaf harvesting can be effectively conducted at 60 days of cultivation, as the additional growth observed at 90 days does not guarantee extending the cultivation period.

## Figures and Tables

**Table 1:** Enzymatic activity (mg p-nitrophenol kg soil h^−1^) of the substrates used after 90 days of *Mentha piperita* cultivation.

Substrate	β-glucosidase	Arylsulfatase
CS	25.09 ± 2.70 b *	10.67 ± 0.00 b
SVR	25.49 ± 1.19 b	52.15 ± 4.24 a
SM	63.89 ± 4.65 a	39.91 ± 2.29 a

* Means (±standard error); followed by the same letter in the column do not differ significantly according to Duncan’s test (*p* ≤ 0.05). CS: control group (soil only). SVR: soil, vermicompost, vermiculite, and rock powder. SM: soil with cattle manure.

**Table 2:** Fresh and dry mass (g) of aerial parts and roots of *Mentha piperita* cultivated under different substrates.

Parameter	CS	SVR	SM
Fresh mass aerial parts	58.50 ± 1.95 b *	54.67 ± 1.50 b	92.33 ± 2.33 a
Dry mass aerial parts	18.65 ± 0.62 b	11.95 ± 0.33 c	29.93 ± 0.76 a
Fresh mass roots	350.00 ± 10.81 a	180.25 ± 11.51 b	335.75 ± 21.97 a
Dry mass roots	48.90 ± 3.75 ab	39.22 ± 2.25 b	58.55 ± 4.54 a
Total mass			
Fresh mass	408.50	234.92	428.08
Dry mas	67.55	51.17	88.48
Fresh mass ratio: aerial/root	0.17	0.30	0.27
Dry mass ratio: aerial/root	0.38	0.30	0.51

* Means (±standard error); followed by the same letter in the line do not differ significantly according to Duncan’s test (*p* ≤ 0.05). CS: control group (soil only); SVR: soil, vermicompost, vermiculite, and rock powder; SM: soil with cattle manure.

**Table 3:** Macronutrients (g kg^−1^) in the leaves and roots of *Mentha piperita* cultivated under different substrates.

Treatment	Total N	P	K	Ca	Mg	S
CS leaves	14.05 ± 0.09 b *	2.82 ± 0.14 a	13.45 ± 0.43 b	18.60 ± 0.98 a	7.30 ± 0.20 a	1.16 ± 0.09 a
SVR leaves	14.90 ± 0.75 b	2.46 ± 0.08 a	26.10 ± 0.75 a	18.60 ± 0.87 a	4.45 ± 0.20 c	1.06 ± 0.03 a
SM leaves	20.73 ± 1.42 a	2.93 ± 0.17 a	14.00 ± 0.35 b	15.45 ± 0.43 b	5.53 ± 0.24 b	1.13 ± 0.09 a
CS roots	7.96 ± 0.64 b	0.51 ± 0.01 b	5.72 ± 0.53 c	3.50 ± 0.75 a	1.40 ± 0.12 b	0.30 ± 0.00 a
SVR roots	7.53 ± 0.18 b	0.56 ± 0.04 b	12.23 ± 0.47 a	3.45 ± 0.03 a	2.35 ± 0.14 a	0.20 ± 0.00 a
SM roots	11.45 ± 0.03 a	0.85 ± 0.00 a	8.85 ± 0.26 b	2.95 ± 0.14 a	1.30 ± 0.06 b	0.30 ± 0.00 a

* Means (±standard error); followed by the same letter in the column do not differ significantly according to Duncan’s test (*p* ≤ 0.05) for leaves and roots. CS: control group (soil only); SVR: soil, vermicompost, vermiculite, and rock powder; SM: soil with cattle manure.

**Table 4:** Micronutrients (g kg^−1^) in the leaves and roots of *Mentha piperita* cultivated under different substrates.

Treatment	Cu	Fe	Mn	Zn
CS leaves	8.16 ± 0.58 a *	291.95 ± 26.18 a	211.43 ± 6.18 a	41.66 ± 2.17 a
SVR leaves	8.75 ± 0.26 a	265.76 ± 23.60 a	101.83 ± 1.98 c	47.83 ± 3.30 a
SM leaves	9.00 ± 0.00 a	179.95 ± 1.01 a	170.25 ± 4.01 b	37.36 ± 5.13 a
CS roots	6.50 ± 0.87 b	1503.90 ± 61.26 b	103.25 ± 11.63 c	22.40 ± 1.27 b
SVR roots	16.80 ± 0.17 a	5172.50 ± 7.22 a	164.85 ± 7.19 a	24.65 ± 1.88 ab
SM roots	7.90 ± 0.17 b	1483.60 ± 56.00 b	135.15 ± 1.65 b	29.40 ± 2.31 a

* Means (±standard error); followed by the same letter in the column do not differ significantly according to Duncan’s test (*p* ≤ 0.05). CS: control group (soil only); SVR: soil, vermicompost, vermiculite, and rock powder; SM: soil with cattle manure.

**Table 5:** Chemical composition (%) of *Mentha piperita* essential oil cultivated under different substrates.

Peak	RT	Compound	Ri Calc	Ri Lit	CS	SVR	SM	Reference
1	6.559	(-)β-pinene	941	949	-	-	0.58	[48]
2	8.163	D-limonene	1032	1032	0.70	0.86	6.35	[49]
3	8.174	Eucalyptol	1032	1033	0.46	0.32	1.17	[50]
4	8.234	*Trans*-β-ocimene	1034	1039	0.64	-	0.62	[51]
5	13.037	L-borneol	1161	1166	0.40	-	0.46	[52]
6	14.343	*Trans*-1(7),8-p-menthadien-2-ol	1165	1165	1.00	0.95	-	[53]
7	14.554	*Cis*-dihydrocarvone	1192	1194	0.96	-	2.36	[54]
8	15.657	Dihydrocarveol	1194	1195	34.45	-	-	[55]
9	16.085	Carveol	1222	1225	2.08	1.23	1.78	[56]
10	16.708	Carvone	1248	1242	31.98	51.33	74.18	[57]
11	20.586	Dihydrocarvyl acetate	1339	1344	1.07	0.67	0.72	[58]
12	21.218	Carveol acetate	1355	1345	1.27	1.58	-	[59]
13	22.488	(-)-β-bourbonene	1384	1384	1.84	5.36	2.09	[60]
14	22.817	α-copaene	1391	1394	1.93	0.57	0.26	[51]
15	23.547	β-cubebene	1417	1418	0.18	10.34	3.97	[61]
16	23.884	α-gurjunene	1425	1425	0.14	0.50	-	[62]
17	24.620	β-copaene	1443	1442	1.91	0.56	-	[63]
18	24.891	Caryophyllene	1449	1444	2.03	5.92	2.09	[51]
19	24.896	Humulene	1449	1452	0.88	1.01	0.37	[62]
20	24.980	*Cis*-β-farnesene	1451	1457	1.99	1.75	0.55	[64]
21	25.393	γ-muurolene	1460	1461	-	0.54	-	[65]
22	26.162	Germacrene D	1477	1480	5.39	1.45	0.45	[62]
23	27.296	(+)-*epi*-bicyclosesquiphellandrene	1527	1521	-	3.15	0.81	[66]
24	27.840	α-muurolene	1555	1540	0.15	0.94	-	[67]
25	28.452	*Cis*-calamenene	1564	1566	1.60	2.25	0.74	[68]
26	29.955	(-)-spathulenol	1585	1582	0.76	1.11		[69]
27	30.148	Caryophyllene oxide	1587	1589	0.53	0.76	0.26	[69]
28	32.390	Cubenol	1664	1664	0.95	1.44	1.13	[70]
29	32.449	T-cadinol	1665	1665	0.84	1.26	-	[71]
30	32.903	α-cadinol	1672	1673	1.15	1.70	-	[72]
31	34.052	Aromadendrene oxide-(2)	1679	1678	0.59	-	-	[73]
32	34.076	6-epi-shyobunol	1890	1881	0.54	1.89	0.42	[74]
33	48.170	Phytol	2112	2112	0.17	0.56	-	[75]
		Total Identified			98.58	99.16	98.00	
		Hydrocarbon Monoterpenes			1.34	0.86	5.55	
		Oxygenated Monoterpenes			71.33	53.83	79.95	
		Hydrocarbon Sesquiterpenes			18.04	34.34	9.97	
		Oxygenated Sesquiterpenes			5.36	7.32	1.81	
		Oxygenated Diterpene			0.17	0.56	-	
		Other Compounds			2.34	2.25	0.72	

Compounds listed according to the elution order on the HP-5MS column; Retention Index (RI) calculated using a homologous series of n-alkanes on a capillary column (HP-5MS); Identification based on comparison with mass spectra from the Nist 11.0 library; Area (%): Percentage of the area occupied by the compound within the chromatogram; (-): Compound absent in the sample; RT: Retention time; RI calc: Calculated retention index; RI Lit: Literature retention index; CS: control group (soil only); SVR: soil, vermicompost, vermiculite, and rock powder; SM: soil with cattle manure.

## Data Availability

Data will be made available on request.

## Supplementary Materials

The following supporting information can be downloaded at: https://www.mdpi.com/article/10.3390/plants14182886/s1, Table S1: Soil chemical analysis; Table S2: Soil and substrate analysis used in the cultivation of *Mentha piperita.* Figure S1: Chromatograms and mass spectra of the major compounds identified in the CS treatments.
