# Supplementary material for: Substrates in Organic Mint Cultivation: Growth, Phytochemistry and Biological Activities
Source: Plants (Basel). 2025 Sep 17;14(18):2886. doi: 10.3390/plants14182886 (PMC12473572; doi:10.3390/plants14182886)
Supplement: Supplementary file 1 [file plants-14-02886-s001.zip › plants-3820377-supplementary.pdf]

Substrates in organic mint cultivation: growth, phytochemistry and biological activities

Supplementary material

Table S1. Soil chemical analysis

Parameter	Unit of Measurement	Analysis Result
P	mg dm^{-3}	69.30
C	g dm^{-3}	6.43
Organic matter	g dm^{-3}	11.08
pH ($CaCl_2$)	-	5.80
Al^{3+}	cmol dm^{-3}	0.00
Potential Acidity	cmol dm^{-3}	2.19
Ca^{2+}+Mg^{2+}	cmol dm^{-3}	0.88
Ca^{2+}	cmol dm^{-33}	0.63
Mg^{2+}	cmol dm^{-3}	0.25
P^{+}	cmol dm^{-3}	0.23
Sum of Bases	cmol dm^{-3}	1.11
C.E.C	cmol dm^{-3}	3.30
Base Saturation	%	33.55
Calcium Saturation	% of Exchange Complex	18.96
Magnesium Saturation	% of Exchange Complex	7.59
Potassium Saturation	% of Exchange Complex	7.00
Ca/Mg	Ratio	2.50
Ca/K	Ratio	2.71
Mg/K	Ratio	1.08

Phosphorus (P). Carbon (C). Aluminum (Al^{3+}). Calcium + Magnesium (Ca^{2+} + Mg^{2+}). Calcium (Ca^{2+}). Magnesium (Mg^{2+}). Potassium (K^{+}). Cation Exchange Capacity (CEC). Calcium to Magnesium ratio (Ca^{2+}/Mg^{2+}). Calcium to Potassium ratio (Ca^{2+}/K^{+}). Magnesium to Potassium ratio (Mg^{2+}/K^{+}).

Table S2. Soil and substrate analysis used in the cultivation of *Mentha piperita*.

DETERMINATIONS	CS	DETERMINATIONS	SVR dry basis 65 °C	SVR wet basis	SM dry basis 65 °C	SM wet basis
pH $CaCl_2$	6.52	pH ($CaCl_2$ 0.01 mol L^{-1})		6.40		6.60
Col OM (g dm^{-3})	12.7	Density		0.78 g cm^{-3}		0.97 g cm^{-3}
P Res (mg dm^{-3})	213.3	MT 65 °C		24.91		7.49
Ca KCl 1 mol L^{-1} (mmolc dm^{-3})	33.1	MT 110 °C		0.13		0.43
Mg KCl 1 mol L^{-1} (mmolc dm^{-3})	15.9	MT total		25.04		7.92
K Res (mmolc dm^{-3})	0.79	OM (%)	2.52	1.89	5.68	5.25
H+Al SMP (mmolc dm^{-3})	10.2	OC (%)	1.40	1.05	3.16	2.92
SB (mmolc dm^{-3})	49.8	IMR (%)	91.10	68.41	88.06	81.46
CEC (mmolc dm^{-3})	60	MR (%)	6.61	4.96	6.01	5.56
V (%)	83	TMR (%)	97.71	73.37	94.07	87.02
		Total N (%)	0.20	0.15	0.34	0.31
		Total P_2O_5 (%)	0.32	0.24	0.30	0.28
		Total K_2O (%)	0.03	0.02	0.03	0.03
		Total Ca (%)	0.44	0.33	0.40	0.37
		Total Mg (%)	1.11	0.83	0.32	0.30
		Total SO_4 (%)	0.08	0.06	0.13	0.12
		C/N Ratio		07.00		09.00
		EC (mS cm^{-1})		0.11		0.33

CS: corrected soil; SVR: soil + vermicompost + vermiculite + rock powder. SM: soil + cattle manure. pH($CaCl_2$ 0.01 mol L^{-1}); Density; MT 65 °C: Moisture 65 °C; MT 110 °C: Moisture 110 °C; MT Total: Total moisture; MO: Organic matter ; OC: Organic Carbon; IMR / MR / TMR: Insoluble Mineral Residue, Mineral Residue, and Total Mineral Residue; N Total: Total Nitrogen; Total P_2O_5: Total phosphorus; Total K_2O: Potassium Total; Total Ca: Total: Total calcium; Total Mg: Total Magnesium; Total SO_4: Total sulfur; C/N Ratio: Carbon to nitrogen ratio; EC: Electrical conductivity; Col OM: Organic matter determined by colorimetric method; P Res: phosphorus resin; Ca KCl: Calcium KCl 1 mol L^{-1}; Mg KCl: Magnesium KCl 1 mol L^{-1}; K Res: potassium resin; H+Al SMP: Potential acidity extracted with SMP buffer; SB: Sum of exchangeable bases; CEC: Cation Exchange Capacity; V: Base saturation percentage.

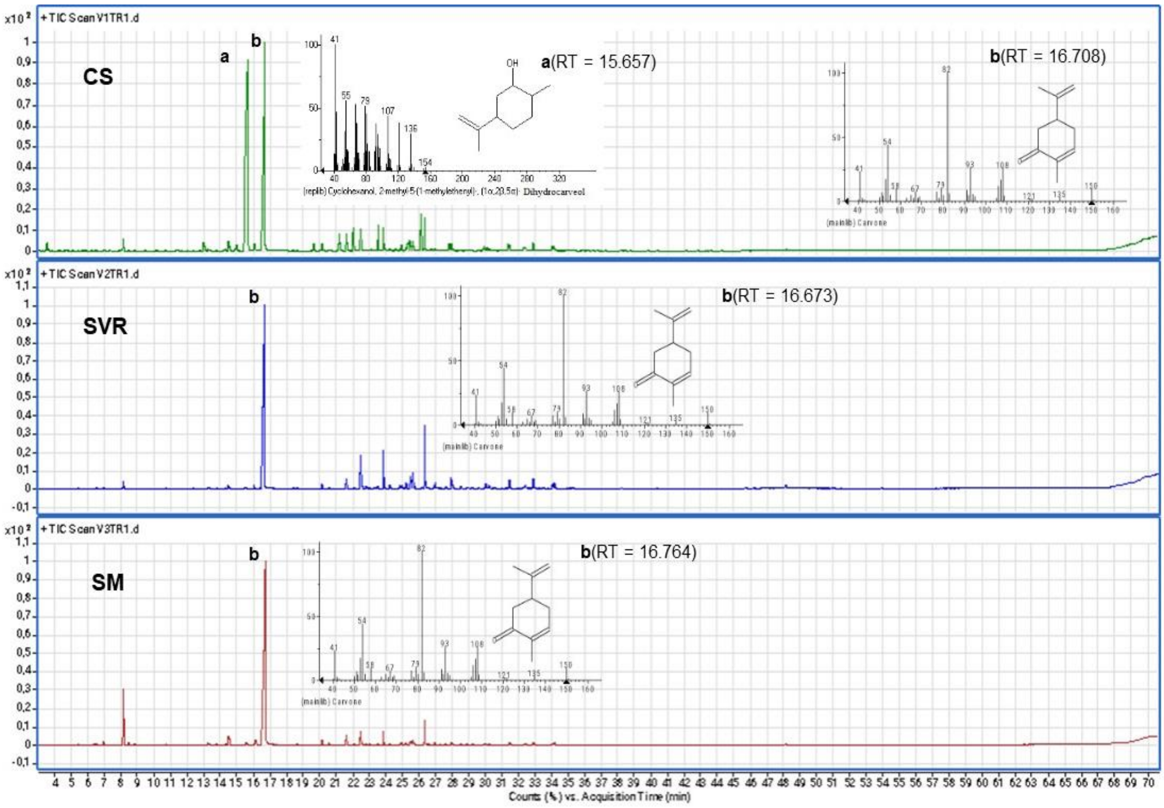

Figure S1: Chromatograms and mass spectra of the major compounds identified in the CS treatments: control group (soil only), a: dihydrocarveol and b: carvone; SVR (soil, vermicompost, vermiculite, and rock powder), b: carvone; SM (soil with cattle manure), b: carvone.
